# Clostridium septicum infection and hemolytic uremic syndrome.

**DOI:** 10.3201/eid0402.980224

**Published:** 1998

**Authors:** M Barnham, N Weightman

**Affiliations:** Harrogate General Hospital, United Kingdom.

## Abstract

Five cases of Clostridium septicum infection secondary to Escherichia coli O157-induced hemolytic uremic syndrome have been reported. We report on three cases (one of which is included in the above five) of dual Cl. septicum and E. coil infection; all three patients were exposed to farm animals. A common zoonotic source for Cl. septicum and E. coli O157 infections should be considered. Patients with hemolytic uremic syndrome should be treated aggressively and monitored closely for Cl. septicum superinfection.


### *Clostridium septicum* Infection and Hemolytic Uremic Syndrome

**M. Barnham* and N. Weightman†**
*Harrogate General Hospital, Harrogate, United Kingdom; and
†Northallerton Friarage Hospital, North Yorkshire, United Kingdom

Five cases of *Clostridium septicum* infection secondary to *Escherichia coli* O157–induced hemolytic uremic syndrome have been reported. We report on three cases (one of which is included in the above five) of dual *Cl. septicum* and *E. coli* infection; all three patients were exposed to farm animals. A common zoonotic source for *Cl. septicum* and *E. coli* O157 infections should be considered. Patients with hemolytic uremic syndrome should be treated aggressively and monitored closely for *Cl. septicum* superinfection.

Severe infection with *Clostridium septicum* in humans is relatively rare. In recent years, the annual incidence of reported bacteremic infection in England and Wales has been 0.4 to 1.0 cases per million population, although compared with other clostridial bacteremias, *Cl. septicum* infection has been second only to *Cl. perfringens* (Public Health Laboratory Service, Communicable Disease Surveillance Centre, Colindale: pers. comm.).

In 1877, *Cl. septicum*, then known as the "vibrion septique," was the first pathogenic anaerobe cultured by Pasteur and Joubert (1). The organism reportedly caused much of the gas gangrene in wounded soldiers during the First and Second World Wars (2), although the bacteriologic techniques of the time were primitive, and findings varied. In addition to gas gangrene, *Cl. septicum* can cause other severe focal or disseminated infections by spontaneous invasion from the gut of compromised patients (3,4). Strong associations have been established between these spontaneous forms of infection and colonic malignancy (especially in the cecum) (5), acute leukemia or cyclical neutropenia (with the development of neutropenic enterocolitis) (6,7), and diabetes. The organism may be more likely than other clostridia to establish infection in viable tissues by virtue of its aerotolerance and ability to establish infection (at least in animal models) from a small inoculum (4).

Address for correspondence: M. Barnham, Department of Microbiology, Harrogate General Hospital, Knaresborough Road, Harrogate, United Kingdom HG2 7ND; fax: 44-1423-883-391.

We have diagnosed serious *Cl. septicum* infection (three bacteremic) in four English patients in a period of 13 years. One of these infections developed as a complication of *Escherichia coli* O157-induced hemolytic uremic syndrome (HUS); with four similar case reports (8-11), this condition appears to be emerging as one with a high risk of *Cl. septicum* superinfection. Details of five published reports of HUS-associated invasive *Cl. septicum* infection are shown in the Table. Mucosal damage and thrombocytopenia from, respectively, Shiga toxin-producing *E. coli* enteritis and HUS appear to provide highly suitable conditions for invasion by *Cl. septicum* in infected patients (5). Caya et al. (7) speculate that thrombocytopenia may impair endothelial resistance to clostridial invasion, and there is gathering evidence for the importance of platelets in host defense against infection (12). When antibiotics are not given, as in the management of *E. coli*-associated HUS, clostridia have more opportunity to thrive unhindered. The five patients in this case sequence did not show granulocytopenia, a feature thought to potentiate invasive clostridial infection in those with neutropenic enterocolitis (6) and in leukemic patients (7); in the latter group, the thrombocytopenia that commonly coexists with neutropenia might be relevant in the evolution of infection. Four of the five patients died, and the three examined at autopsy had colonic ulceration and hemorrhagic necrosis. Unusual clinical features included intracranial infection with *Cl. septicum* in four and clostridial cellulitis in the abdominal wall in two.

Table. Hemolytic uremic syndrome–associated *Clostridium septicum* infection

| Country, year (ref) | Sex,[a] age (yrs) | *E. coli*[b] in feces | *Cl. septicum* isolated from | Cytopenias[c] granulo- | Cytopenias[c] thrombo- | Clinical features—outcome |
|---|---|---|---|---|---|---|
| USA, 1988 (8) | M, 1 | NS[d] | Rash aspirate, PM[e] blood | No | Yes | Abdominal rash, sepsis, convulsions, cerebritis, necrosis colon—died |
| England, 1993 (9) | M, 4 | O157 | Blood, PM brain | No | Yes | Convulsions, cerebritis, necrosis colon—died |
| USA, 1993 (10) | F, 2 | *E. coli* | Blood, cerebrospinal fluid | No | Yes | Sepsis, cerebritis, meningitis, necrosis colon—died |
| USA, 1995 (11) | M, 2 | O157 | Abscess pus | No | Yes | Brain abscess—recovered |
| England, 1997 (this study) | M, 2 | O157 | Blood | No | Yes | Septic shock—died |

[a] M, male; F, female.
[b] *Escherichia coli*.
[c] At HUS = at the time that hemolytic uremic syndrome was evident.
[d] NS, not stated.
[e] PM, postmortem.

Both *Cl. septicum* and *E. coli* O157 have herbivore animal reservoirs. We propose as a possible sequence of events for our three cases a) acquisition of the organisms from animals, b) colonization of the colon of the patient, leading to c) invasion by *Cl. septicum* as described above. Two of three cases of serious *Cl. septicum* infection also were linked to sheep; the remaining case, in an elderly man from a suburban setting, who had carcinoma of the cecum and *Cl. septicum* septicemia, resembles the typical case descriptions recorded by others (3,5).

In May 1997, a 2-year-old boy was hospitalized with a 7-day history of vomiting and blood-flecked watery diarrhea; *E. coli* O157 phage-type 1, VT-type 1+2 had been isolated from fecal specimens. On admission, the patient was pale and uncomfortable with a petechial rash on the trunk and an oral temperature of 38.2°C; the abdomen was soft with no tenderness, and blood pressure was normal. Investigations showed hemoglobin 9.5 g/dL with some burr cells and microspherocytes present, elevated total white cell count, and thrombocytopenia. Renal function was impaired, with blood urea elevated; serum creatinine was within the reference range. Blood cultures were taken on admission. After diagnosis of HUS, the patient was given intravenous fluid replacement and was transferred to the local teaching hospital where blood cultures were repeated and antibiotics were initially withheld.

Peritoneal dialysis initially produced clinical improvement, but 32 hours after admission, the patient suddenly became irritable and confused and had a large hematemesis; his blood pressure fell to 100/40 mm Hg, and he exhibited clinical shock. After transfusion with fresh frozen plasma and albumen, cefuroxime, and metronidazole, he was transferred to the intensive care unit, intubated, and ventilated. The pulse rate rose to 163/min and the circulating white blood cell count to 34.0 x $10^9$/L. The abdomen became distended, and at the peritoneal dialysis catheter entry site, an area of superficial skin ischemia resembling fasciitis developed, which later progressed over the right side of the abdomen. A plain radiograph showed dilated loops of bowel with thickened walls but no free gas. A diagnosis of ischemic bowel was made, but 8 hours after his sudden deterioration and before an exploratory laparotomy could be performed, the patient went into cardiorespiratory arrest and died. Both sets of blood cultures yielded *Cl. septicum*, the first becoming positive after 48 hours of incubation. An autopsy was not performed.

The boy had played on a dairy and sheep farm, where *E. coli* O157 with identical banding on digested DNA pulsed-field gel electrophoresis analysis to that from the boy was isolated from five cows, but no search was made for *Cl. septicum*.

A 64-year-old hiker in August 1984 slipped against a rock on a sheep trail and cut her leg a few centimeters above the ankle. Emergency treatment consisted of wound suturing and a tetanus toxoid booster. Three days later, the patient returned to the Emergency Department flushed and sweating with a grossly swollen, discolored, blistered, and foul-smelling leg. The limb was clearly gangrenous to the lower third of the thigh with patchy erythema and edema in the buttock and lower abdominal wall; radiographs showed large quantities of gas in the soft tissues of the leg. Findings included an oral temperature of 38.8°C, blood pressure 100/60 mm Hg, hemoglobin 14.1 g/dL, white blood cell count 5.3 x $10^9$/L (including lymphopenia of 0.58 x $10^9$/L), and thrombocytopenia (platelets 101 x $10^9$/L). *Cl. septicum* was isolated in pure culture from blood as well as from swabs of blisters on the leg, where it was found together with *E. coli* and *Bacillus cereus*.

The patient's leg was amputated mid thigh, and she received gas gangrene antiserum, blood transfusion, and intravenous benzyl penicillin and metronidazole. Postoperative complications included hypotension, atrial fibrillation, and acute renal failure, but she responded to treatment and recovered.

A 71-year-old male, sheep-farm laborer was cleaning out sheep dipping pits in September 1985 when he caught his arm in the slurry machinery, causing a traumatic amputation above his elbow joint with dislocation of the head of the humerus. Emergency hospital treatment included general resuscitation, blood transfusion, and surgical excision of damaged bone and soft tissues together with reduction of the dislocated shoulder.

Despite postoperative treatment with cefuroxime and sodium fusidate, *Cl. septicum*, *E. coli,* and enterococci were repeatedly isolated from the wound; by the sixth postoperative day, a copious and very offensive watery discharge exuded from the wound, with crepitus under the edges. At this time the patient's general condition remained good except for an oral temperature of 38.5°C and circulating white cell count of 16.5 x $10^9$/L; platelet counts remained within the normal range. Surgical exploration revealed local necrosis of soft tissues in the wound down to the bone; devitalized tissues were excised, and drainage was promoted. Intravenous benzyl penicillin, ampicillin, and metronidazole led to rapid resolution of infection and a good recovery.

Opinions differ as to whether *Cl. septicum* is part of the normal human fecal flora (5,13). Studies have reported the presence of the organism in fecal specimens of 2% to 3% of healthy persons consuming relatively large quantities of beef (14), but it is uncertain whether the organism was resident or transient. Earlier studies (14) involving very small numbers of healthy persons are unconvincing. Human infection with *Cl. septicum* is assumed to be essentially autogenous, and the possibilities of environmental or foodborne exposure originating from animals have not been adequately discussed. *Cl. septicum* is found in the feces of herbivores, and it is readily cultivated from soil in areas where they graze; the dominant causative clostridium in malignant edema (posttraumatic gangrene) in domestic animals, it causes a form of necrotizing enteritis (braxy) in young sheep grazing on frozen vegetation during the autumn and winter months (15).

The three cases described above suggest an infection of animal origin. In the first case, *E. coli* O157 was almost certainly acquired by the young boy from a farm contaminated by sheep and cattle, suggesting coexposure to clostridia. The hiker's wound was probably contaminated with spores from an area of intensive sheep grazing; Northern England is considered an area of high risk for *Cl. septicum* infection in sheep (16). In the third case, organisms from the slurry or sheep dip were probably injected directly into the wound; sheep dips can become highly contaminated with *Cl. septicum*, causing high incidence of infection (15).

We need to learn more about the role of animals in the epidemiology of colonization and disease with *Cl. septicum* in humans. *E. coli* O157 and associated HUS appear to be increasing in many parts of the industrialized world; severe *Cl. septicum* infection can occur as a secondary disease. The occurrence of at least five such dual infections suggests important elements linking the evolution and pathogenesis of the two infections; furthermore, acquisition of both organisms from a common zoonotic source remains a possibility. A high level of suspicion for *Cl. septicum* superinfection in patients with HUS, rapid diagnosis, prompt antimicrobial therapy, and urgent surgical attention, as required, can improve the survival rate of patients with this life-threatening infection.

### References

1. Sebald M, Hauser D. Pasteur, oxygen and the anaerobes revisited. Anaerobe 1995;1:11-6.

2. MacLennan JD. Histotoxic clostridial infections of man. Bacteriology Reviews 1962;26:176-276.
3. Koransky JR, Stargel MD, Dowell VR. *Clostridium septicum* bacteremia—its clinical significance. Am J Med 1979;66:63-6.
4. Stevens DL, Musher DM, Watson DA, Eddy H, Hamill RJ, Gyorkey F, et al. Spontaneous, nontraumatic gangrene due to *Clostridium septicum*. Reviews of Infectious Diseases 1990;12:286-96.
5. Kornbluth AA, Danzig JB, Bernstein LH. *Clostridium septicum* infection and associated malignancy. Medicine 1989;68:30-7.
6. *Clostridium septicum* and neutropenic enterocolitis. Lancet 1987;2:608.
7. Caya JG, Farmer SG, Ritch PS, Wollenberg NJ, Tieu TM, Oechler HW, et al. Clostridial septicemia complicating the course of leukemia. Cancer 1986;57:2045-8.
8. Riccio JA, Oberkircher OR. *Clostridium septicum* sepsis and cerebritis: a rare complication of the hemolytic-uremic syndrome. Pediatr Infect Dis J 1988;7:342-5.
9. Randall JM, Hall K, Coulthard MG. Diffuse pneumocephalus due to *Clostridium septicum* cerebritis in hemolytic uremic syndrome. Neuroradiology 1993;35:218-20.
10. Broughton RA, Lee EY. *Clostridium septicum* sepsis and meningitis as a complication of the hemolytic-uremic syndrome. Clin Pediatr (Phila) 1993;32:750-2.
11. Chiang V, Adelson PD, Poussaint TY, Hand M, Churchwell KB. Brain abscess caused by *Clostridium septicum* as a complication of hemolytic-uremic syndrome. Pediatr Infect Dis J 1995;14:72-4.
12. Yeaman MR. The role of platelets in antimicrobial host defense. Clin Infect Dis 1997;25:951-70.
13. Lorber B. Gas gangrene and other clostridium-associated diseases. In: Mandell GL, Bennett JE, Dolin R, editors. Principles and practice of infectious diseases. 4th ed. New York: Churchill Livingstone; 1995. p. 2182-95.
14. George WL, Finegold SM. Clostridia in the human gastrointestinal flora. In: Borriello SP, editor. Clostridia in gastrointestinal disease. Boca Raton (FL): CRC Press; 1985. p. 1-37.
15. Radostits OM, Blood DC, Gay CC. Veterinary medicine: a textbook of the diseases of cattle, sheep, pigs, goats and horses. 8th ed. London: Bailliere Tindall; 1994. p. 686-9.
16. Martin WB, Aitken ID. Diseases of sheep. 2nd ed. Oxford: Blackwell Scientific Publications; 1991. p. 109-13.
